# Frontoparietal tDCS Benefits Visual Working Memory in Older Adults With Low Working Memory Capacity

**DOI:** 10.3389/fnagi.2018.00057

**Published:** 2018-03-13

**Authors:** Hector Arciniega, Filiz Gözenman, Kevin T. Jones, Jaclyn A. Stephens, Marian E. Berryhill

**Affiliations:** ^1^Memory and Brain Laboratory, Department of Psychology, Program in Cognitive and Brain Sciences, and Integrative Neuroscience, University of Nevada, Reno, NV, United States; ^2^Department of Psychology, Yaşar University, İzmir, Turkey; ^3^Department of Psychology, Colorado State University, Fort Collins, CO, United States; ^4^Department of Occupational Therapy, Colorado State University, Fort Collins, CO, United States

**Keywords:** working memory, tDCS, aging, visual working memory, visual working memory capacity

## Abstract

Working memory (WM) permits maintenance of information over brief delays and is an essential executive function. Unfortunately, WM is subject to age-related decline. Some evidence supports the use of transcranial direct current stimulation (tDCS) to improve visual WM. A gap in knowledge is an understanding of the mechanism characterizing these tDCS linked effects. To address this gap, we compared the effects of two tDCS montages designed on visual working memory (VWM) performance. The bifrontal montage was designed to stimulate the heightened bilateral frontal activity observed in aging adults. The unilateral frontoparietal montage was designed to stimulate activation patterns observed in young adults. Participants completed three sessions (bilateral frontal, right frontoparietal, sham) of anodal tDCS (20 min, 2 mA). During stimulation, participants performed a visual long-term memory (LTM) control task and a visual WM task. There was no effect of tDCS on the LTM task. Participants receiving right unilateral tDCS showed a WM benefit. This pattern was most robust in older adults with low WM capacity. To address the concern that the key difference between the two tDCS montages could be tDCS over the posterior parietal cortex (PPC), we included new analyses from a previous study applying tDCS targeting the PPC paired with a recognition VWM task. No significant main effects were found. A subsequent experiment in young adults found no significant effect of either tDCS montage on either task. These data indicate that tDCS montage, age and WM capacity should be considered when designing tDCS protocols. We interpret these findings as suggestive that protocols designed to restore more youthful patterns of brain activity are superior to those that compensate for age-related changes.

## Introduction

Visual working memory (VWM) allows us to integrate perception across interruptions such as eye movements. As we age, working memory (WM) performance, including VWM, shows decline (Reuter-Lorenz and Sylvester, 2005; Iachini et al., 2009; Craik et al., 2010). Accompanying performance changes are a number of changes in functional brain activations. For example, during many cognitive tasks it is observed that there is a posterior-anterior shift in aging (PASA; Davis et al., 2008), and a reduced specialization of brain networks (Chan et al., 2014). The PASA pattern generalizes across a range of cognitive functions including attention (Madden et al., 2002; Cabeza et al., 2004; Ansado et al., 2012), visual perception (Grady, 1996, 2000; Iidaka et al., 2002; Ansado et al., 2012), visuospatial processing (Nyberg et al., 2003; Meulenbroek et al., 2004; Ansaldo et al., 2015), VWM (D’Esposito et al., 2000; Grossman et al., 2002; Dennis and Peterson, 2012; Jockwitz et al., 2017), and episodic memory (Anderson et al., 2000; Cabeza et al., 2004; Gutchess et al., 2005; Dennis and Peterson, 2012). In older adults there is reduced hemispheric asymmetry for tasks typically right or left lateralized in the young (Cabeza, 2002). Thus, compared to younger adults, older adults tend to have greater bilateral frontal activity that is less lateralized as a function of task demands (see also Rypma and D’Esposito, 2000; Schneider-Garces et al., 2010). In short, in studies comparing the functional activation patterns of young and old adults a more lateralized, unilateral pattern becomes more bilateral and frontal with age.

The functional importance of these changes in patterns of neural activity pattern remains a topic of debate. Two views guiding our thinking are the perspectives put forth in the compensation-related utilization of neural circuits hypothesis (CRUNCH: Dennis et al., 2008; Reuter-Lorenz and Park, 2014), and the scaffolding theory of aging and cognition (STAC: Park and Reuter-Lorenz, 2009). These views suggest that older adults recruit greater bilateral frontal activations to maintain cognitive performance but that this compensation is finite (Reuter-Lorenz and Cappell, 2008; Carp et al., 2010). It is finite in the sense that older adults with lower cognitive ability are likely to draw on and exhaust compensation at lower levels of task difficulty than those with stronger cognitive ability (Schneider-Garces et al., 2010). In other words, older adults use their brains differently and predictably than younger adults to perform VWM tasks, and the effectiveness of this alternative usage is limited by an individual’s capacity.

Furthermore, a right-lateralized, unilateral pattern of brain activity is apparent when younger adults perform a VWM task. Functional magnetic resonance imaging (fMRI) studies report that right posterior parietal cortex (PPC) activity reflects VWM load showing increases in BOLD signals until memory capacity plateaus (Cabeza and Nyberg, 2000; Todd and Marois, 2004, 2005; Xu and Chun, 2006). Similarly, event-related potentials (ERPs) reveal sustained negativity over posterior electrodes correlating with VWM load (Vogel and Machizawa, 2004; Vogel et al., 2005). Neuropsychological participants with parietal lobe lesions show selective deficits in VWM tested by recognition probes (Berryhill and Olson, 2008a,b; Olson and Berryhill, 2009; Berryhill, 2012). Transcranial direct current stimulation (tDCS) studies targeting PPC regions have also found that anodal tDCS can improve VWM (Sandrini et al., 2011; Jones and Berryhill, 2012; Tseng et al., 2013; Juan et al., 2017), or interfere with VWM (Berryhill et al., 2010; Jones and Berryhill, 2012). Overall, these findings point toward a functional contribution of the PPC to VWM.

These changing patterns of activation raise the following questions: is it advantageous to enhance bilateral frontal regions in older adults? Or, is it be superior to reinforce patterns of brain activity that are more lateralized? One way to test this question is to apply noninvasive stimulation such as tDCS. tDCS shows promise because it is well-tolerated, safe and affordable. Importantly, in our recent longitudinal studies pairing tDCS with VWM tasks reveal improvements in VWM performance in the healthy aging population (Jones et al., 2015b; Stephens and Berryhill, 2016). These studies indicated that multiple sessions targeting frontal or frontoparietal regions effectively strengthen trained task performance and performance on untrained transfer tasks, both near (Richmond et al., 2014; Jones et al., 2015b), and far transfer (Stephens and Berryhill, 2016). Studies in other cognitive domains have also found that multiple sessions extend benefits on executive functions (Gill et al., 2015; Au et al., 2016), language (Cotelli et al., 2014), and VWM performance in patient populations (Park et al., 2003; Jo et al., 2009; Park and Gooding, 2014; Wu et al., 2016) thereby demonstrating the feasibility and potential value of tDCS-linked cognitive training approaches.

A challenge in the tDCS literature is the incomplete understanding of the mechanism by which tDCS elicits cognitive benefits. This is exacerbated by the lack of consistency in tDCS protocols and VWM tasks. In VWM, tDCS is most consistently successful when applied over frontal sites (Berryhill et al., 2014; Wu et al., 2014), however; protocols differ across groups. More problematically, a growing literature shows that individual differences are tremendously important in predicting benefit or impairment after the same protocol. Several studies report that individual differences predict different responses to a single session of tDCS during VWM task performance (Berryhill et al., 2014; Li et al., 2015; London and Slagter, 2015; Hsu et al., 2016; Puri et al., 2016; Juan et al., 2017; Katz et al., 2017). For instance, we found that participants with more education or higher VWM capacity improved after a session of tDCS whereas less well-educated or low VWM participants do not (Berryhill and Jones, 2012; Jones et al., 2015a; for a recent review see Berryhill, 2017). This contributes to variability and the percept that tDCS is not effective for studying cognitive questions (Horvath et al., 2015b).

Our goal was to test whether frontoparietal or bifrontal tDCS montages would show differential benefits on VWM in healthy older adults. We also compared performance as a function of WM capacity, based on our previous findings. To clarify the interpretation of these initial findings, we included new analyses from an experiment in which the right PPC alone had been targeted by tDCS (Experiment 1B). Furthermore, we were interested in determining whether these effects would generalize to young adults who already perform WM tasks at a superior level and rely on unilateral brain activation patterns. We completed a second experiment in young adults (Experiment 2).

## Experiment 1A: Healthy Older Adults

Here, we tested whether a montage designed to facilitate neural compensation or restoration was better at enhancing VWM performance in low and or high VWM capacity older adults. Regardless of the outcome, these data would be useful in tailoring tDCS protocols to maximize benefit. This answer may be important for enhancing the consistency of tDCS outcomes and for understanding the underlying mechanism of tDCS effects.

## Materials and Methods

### Participants

Our previous data indicate that an effect size of ~0.51 can be anticipated. To preserve power = 0.95, *α* = 0.05 and estimating a modest effect size (partial *η*^2^ = 0.21, Jones and Berryhill, 2012), there were 36 self-reported right-handed older adults (mean age: 67.72 years, standard deviation (SD): 4.52, 20 females, mean education: 16.11 years (SD): 1.8) participants. The study was single-blinded (participants were not aware of the hypotheses tested and did not know which tDCS condition they experienced each session). Participants were screened to ensure no one had a history of neurological or psychiatric disorders or head injuries, and that they were not taking prescriptions for neuroleptic, hypnotic, or anti-seizure medications. Older adults scoring <26 on the Montreal Cognitive Assessment (MOCA; version 7.1) were excluded to ensure that participants were unlikely to have cognitive impairment (Pendlebury et al., 2015). Five participants were excluded after screening. All procedures described in this article were approved and conducted in accordance with the University of Nevada Institutional Review Board. Participants signed informed consent documents and received $15/h.

### Spatial Span Task: Corsi Blocks

To obtain an independent baseline measure of visuospatial VWM capacity, participants completed the Corsi Blocks Spatial Span task from the MATRICS Consensus Cognitive Battery (Nuechterlein et al., 2008). Participants repeated a sequence of block taps that lengthened. The sequence length incremented after two trials of a given length and the task ended when the participant failed two trials at a given sequence length. The Corsi Block Spatial Span score indicated the largest sequence length the participant could repeat correctly. The Corsi Block Spatial Span task was conducted on the first day prior to any stimulation and VWM tasks.

### Experimental Tasks

Two experimental tasks were used, a long-term memory (LTM) task and a VWM task. The LTM task served as a control task because it was not expected to change as a function of tDCS so that it could confirm the limited nature of tDCS-linked benefits for each montage. The VWM task was the experimental task of greatest interest and followed our line of work showing tDCS-linked differential performance.

### Long-Term Memory Task

**Figure 1:**
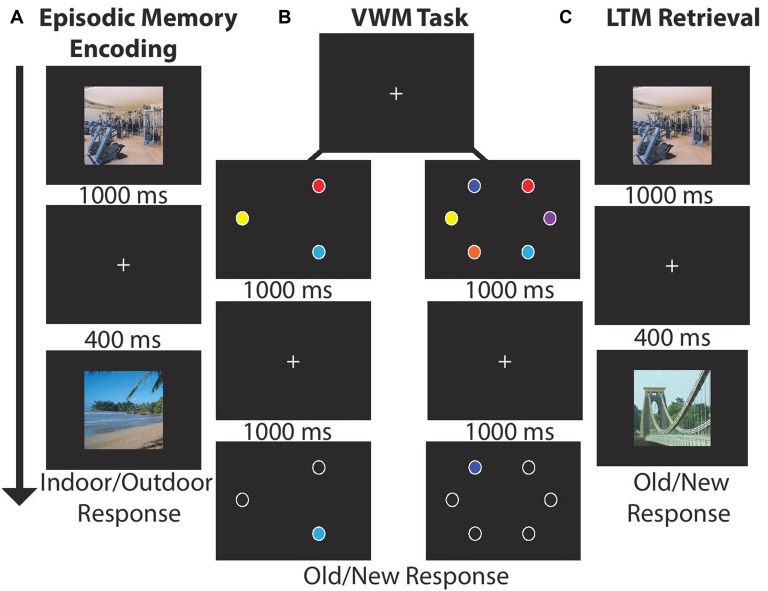
Experimental paradigm for each block. **(A)** Long-term memory (LTM) task: participants viewed 15 images (1000 ms) and indicated whether the scene was indoor/outdoor (50% each). Each image was followed by a by delay (400 ms). After encoding participants completed a visual working memory (VWM) task. **(B)** VWM task: participants viewed stimulus arrays (1000 ms) consisting of either three or six stimuli followed by a delay (1000 ms). Next, a single probe item appeared and participants made a speeded old/new response (unlimited response time). **(C)** LTM retrieval: participants viewed 30 scenes and judged whether the item was old (50%) or new.

Participants sequentially viewed 15 centrally located scenes (1000 ms, 400 ms ISI, 21° × 17°) and made an indoor/outdoor (50%) judgment via button press response. LTM was tested in a recognition paradigm conducted after the VWM task. Participants indicated which of 30 scenes were old (50% old). There were four blocks of LTM task trials for a total of 120 recall trials; see Figures 1A,C.

### Working Memory Task

Participants viewed stimulus arrays containing three (50%) or six colored circles (1000 ms, 4.7° × 4.7°, 5° eccentricity) drawn from a set of nine color patches. After a delay (1000 ms) one probe item appeared, and participants made a unspeeded old/new item-location recognition response via button press. In each block of trials participants completed 24 trials per set size and four total blocks for a total of 192 VWM trials; see Figure 1B. The task took ~25 min.

### Transcranial Direct Current Stimulation

tDCS was applied via a battery-driven constant current stimulator (Eldith Magstim, GmbH, Ilmenau, Germany). The same participants completed three counterbalanced tDCS sessions on different days separated by at least 24 h. Participants were also blinded to the nature of the stimulation. In the *unilateral* session, the anode was placed over right PFC (F6) and the cathode over right PPC (P6), or *bilateral* PFC-PFC (anode F6, cathode F5), and sham (placebo, counterbalanced assignment to either the unilateral or bilateral montage; Nasseri et al., 2015). TDCS (2 mA, 20 min) was delivered through two 5 × 7 cm^2^ electrodes housed in saline-moistened sponges. Sham stimulation included 20 s of ramping up and down stimulation at the beginning and end to give participants a physical sense of stimulation associated with current change (Gandiga et al., 2006).

### Analysis

High and low VWM capacity groups were formed using a median split on their Corsi Block Spatial Span scores (high: 11.33, low: 8.27, *t*_(34)_ = −8.1, *p* < 0.00001). Due to the median split there were a total of 18 high/low VWM capacity participants. The resulting groups did not differ in terms of age (high: 66.61, low: 68.83, *p* = 0.14), or MOCA (high: 27.61, low: 27.38, *p* = 0.66) scores. To minimize between-subject variability we analyzed normalized difference indices derived from the accuracy data:

Difference Index (DI) = (tDCS % − sham %)/(tDCS % + sham %)

Each participant had a difference index (DI) for each montage and for the VWM set size (3, 6), and for the LTM task. These values were subjected to separate mixed analysis of variance (ANOVA) with the within-subjects factors of stimulation condition (bilateral, unilateral) and the between-subjects factor of VWM capacity (low, high).

## Current Flow Modeling

**Figure 2:**
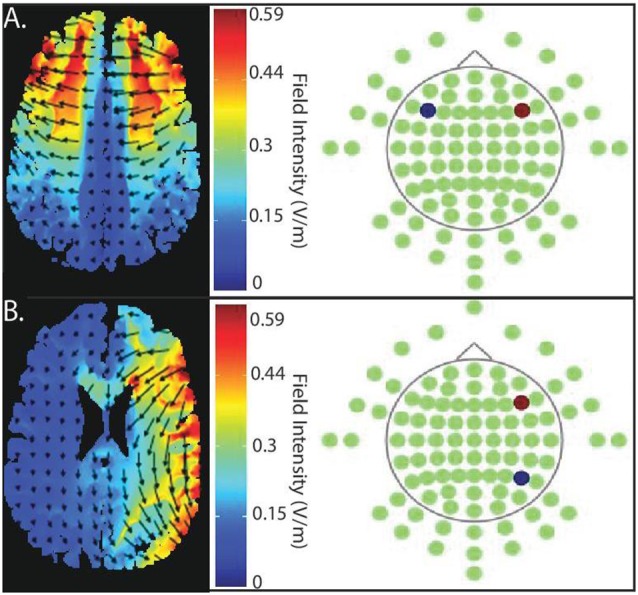
Current flow model of targeted stimulation sites.** (A)** Simulation of bilateral stimulation to prefrontal cortex. Electrodes placed in PFC-PFC (anode (red) placed intermediate between F4 and F8, cathode (blue) placed intermediate between F3 and F7). **(B)** Simulation of unilateral stimulation, which the anode (red) is, situated over right PFC (centered intermediately between F4 and F8) and the cathode (blue) over right posterior parietal cortex (PPC; centered between P4 and P8).

We modeled current flow to demonstrate the extent of current flow and to clarify the region of field intensity across the whole brain using HD-Explore™/tDCS-Explore software (Soterix Medical Inc., New York, NY, USA); see Figure 2.

## Results

### LTM Task Results

To address the specificity of any tDCS-montage effects, we examined LTM performance using a paired sample *t*-test. The normalized LTM DI from unilateral and bilateral showed no significant difference between unilateral (Mean (*M*) = −0.002, SD = 0.04) and bilateral stimulation (*M* = 0.006, SD = 0.05) conditions (*t*_(35)_ = 0.84, *p* = 0.4). There was no effect of tDCS montage on LTM task performance.

### VWM Task Results

**Figure 3:**
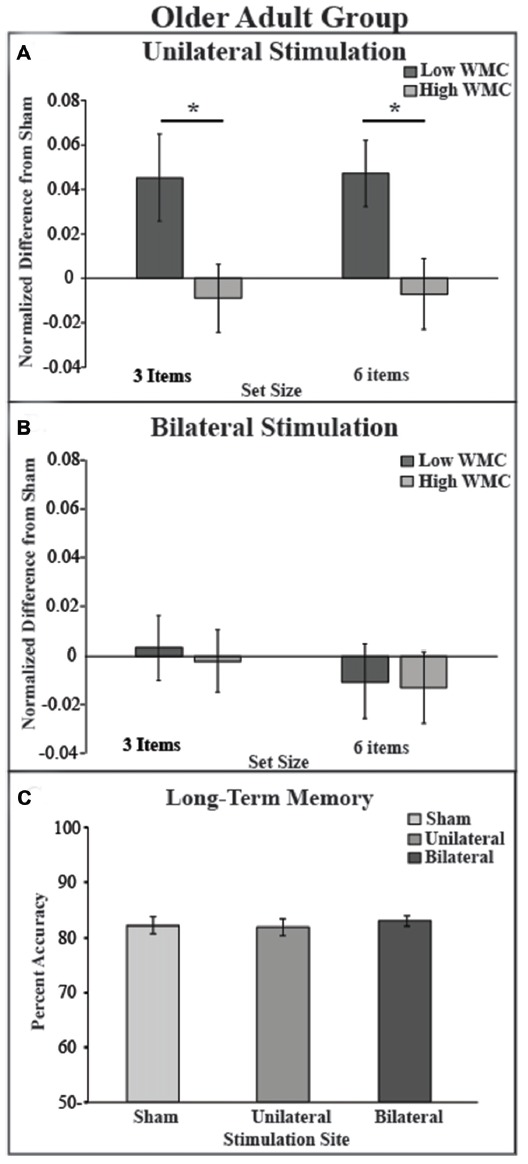
Behavioral outcome by montage and experimental paradigm.** (A)** tDCS to the right unilateral stimulation site improves VWM performance in the low VWM capacity (dark gray bars) but not in the high VWM capacity group (light gray bars). **(B)** tDCS to bilateral prefrontal cortex shows no improvement of VWM in both low and high VWM capacity participants. Performance on the visual VWM task for set size of three is plotted on the left and set size of six is plotted on the right. Values greater than zero reveal tDCS-related improvement in VWM performance. Error bars reflect the standard error. **(C)** In the LTM task there was no main effect of montage. *p < 0.05.

To test whether the tDCS montages differentially modulated VWM performance, DIs were subjected to mixed effects ANOVA with the within-subjects factors of montage (bilateral, unilateral) and set size (3, 6), and the between subject factor of VWM capacity (low, high); see Figure 3. There was a significant main effect of montage (*F*_(1,34)_ = 10.04, *p* = 0.003, partial *η*^2^ = 0.23), such that unilateral stimulation provided greater benefit to VWM than bilateral stimulation. There was also a borderline significant main effect of VWM capacity (*F*_(1,34)_ = 3.6, *p* = 0.06, partial *η*^2^ = 0.096) showing that the low VWM capacity participants received the greatest benefit. Accordingly, there was a significant interaction of montage × VWM capacity (*F*_(1,34)_ = 10.41, *p* = 0.003, partial *η*^2^ = 0.23). This interaction can be explained such that the low VWM capacity participants benefited from unilateral stimulation, whereas the high VWM capacity participants showed little effect of tDCS montage or set size; see Table 1. No other main effects or interactions approached significance (*F*s < 1, *p* > 0.2 for set size, set size × VWM capacity, and montage × set size × VWM capacity, montage × set size).

**Table 1:** Difference indices (DI) and accuracy (%) for each older adult group (low, high working memory (WM) capacity) and set size (3, 6) in the visual working memory (VWM) task.

Measure	Group	Montage	Set size 3	Set size 6	LTM
	Low	Uni	0.04 (0.01)	0.04 (0.04)	0.0027 (0.03)
DI		Bi	0 (0.01)	0.01 (0.01)	0.016 (0.017)
	High	Uni	−0.004 (0.01)	0 (0.04)	−0.006 (0.04)
		Bi	−0.001 (0.01)	−0.01 (0.01)	−0.0018 (0.01)
	Low	Uni	82.5 (0.03)	67 (0.02)	80.4 (0.02)
%		Bi	76.9 (0.02)	61 (0.02)	82.2 (0.01)
		Sham	76.7 (0.03)	62 (0.02)	80 (0.02)
	High	Uni	85.5 (0.03)	67.5 (0.03)	83 (0.02)
		Bi	85.8 (0.03)	66 (0.02)	83.1 (0.01)
		Sham	85.7(0.03)	68 (0.02)	83.7 (0.02)

*Uni, unilateral frontoparietal stimulation; Bi, bilateral frontal stimulation. Means (standard error of the mean)*.

Experiment 1A showed that older adults with low VWM capacity benefited from the unilateral frontoparietal tDCS montage. One possible interpretation of these data is that the unilateral montage serves to restore age-related reductions in frontoparietal networks underlying VWM. Whereas the bifrontal montage did not successfully benefit VWM performance, the role that VWM capacity played in this process remains significant but difficult to predict. Additionally, because the difference between the two montages an alternative interpretation was that the effect was driven by tDCS targeting the right PPC site. In other words, potentially, the benefit was exclusively due to current reaching the PPC, rather than unilateral or bifrontal stimulation. Finally, important to note that behavioral performance did not reach ceiling (>90% accuracy) or floor (below chance) indicating that our task was neither too easy or too difficult; see Table 1.

## Experiment 1B: Healthy Older Adults

To address the concern that tDCS over PPC targets served as the key difference between the two tDCS montages, we include a new analysis from a previously published study (Jones et al., 2015b). The goal of that article was to study longitudinal effects of tDCS paired with VWM training, where we saw lasting benefits across participants, regardless of VWM capacity. Furthermore, one of the active tDCS groups received anodal tDCS over the right PPC. Thus, we can select the sham and PPC data from the first session of tDCS to evaluate VWM tested by recognition, as was done in Experiment 1A. Additional training tasks probing VWM tested by recall were included in the original study, but are not included here. We predicted that if the results from Experiment 1A were attributable to tDCS modulating over the right PPC, then we should observe a significant change in VWM performance when the PPC site alone is targeted. In contrast, if the results were due to conjoint frontoparietal tDCS, then we would not observe a VWM performance change when the right PPC alone is targeted. We might observe a benefit for the high WM capacity participants based on our previous findings showing that highly educated older adults benefited from anodal tDCS to the PPC (Jones and Berryhill, 2012) if education and WM capacity confer similar response to tDCS.

## Materials and Methods

### Participants

In the full study, there were 72 neurotypical self-reporting right-handed older adults who scored a 25 or higher on the Mini-Mental Status Examination (MMSE). Participants were randomly assigned to one of four groups (sham, PFC, PPC, PFC-PPC alternating). Here, we select sham and PPC groups alone for new analysis. Thus, 36 older adults (mean age: 64.52, SD: 5.41, 23 females), divided into two groups similar in age (*p* = 0.83, sham 64.33(5.24), PPC 64.72(5.72)), education (*p* = 0.82, sham 16.72(2.29), PPC 16.94(3.57)), and MMSE score (*p* = 0.58, sham 28.61(1.5), PPC 28.33(1.5)).

### The Automated Operation Span (OSpan)

In the Jones et al. (2015b) study, the OSPAN task was used as an independent measure of VWM capacity instead of the Corsi Block Spatial Span task. The OSpan is a task of divided attention in which participants solve arithmetic problems while simultaneously encoding and maintaining letter strings. Participants are then instructed to recall letters after they have completed the arithmetic problems (Unsworth et al., 2005). The task took ~10 min and consisted of nine sets of letter trials, which ranged from three to seven total letters. Performance was measured by performance on letter recall and math accuracy.

### Experimental Task

**Figure 4:**
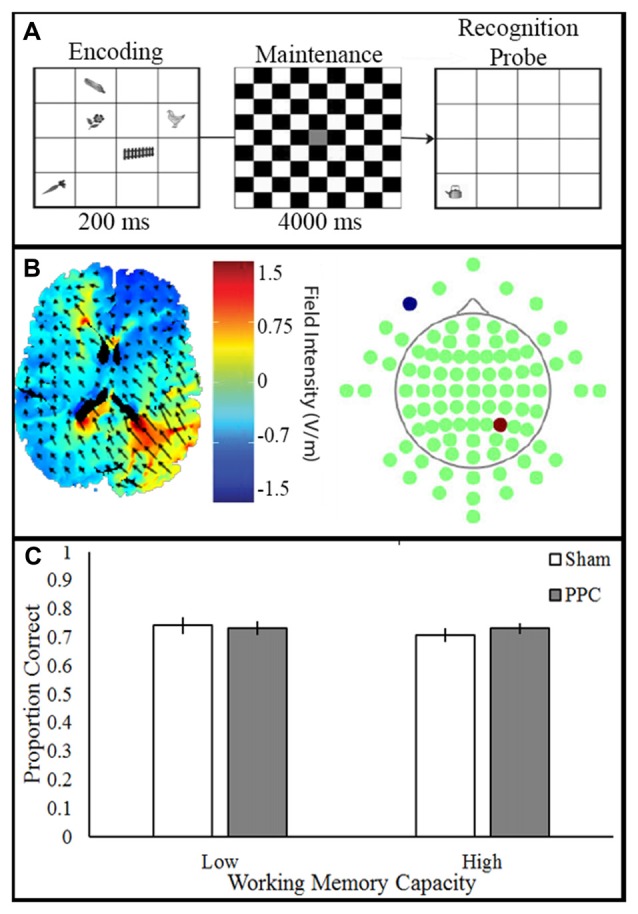
Experimental paradigm, current flow model and behavioral outcome.** (A)** VWM task: participants viewed stimulus array (200 ms) consisting of items followed by a delay (4000 ms). Next, a single probe item appeared and participants made a speeded old/new response (unlimited response time). **(B)** Simulation of right PPC stimulation. Electrodes placed in P4 anode (red) and CC cathode (blue). **(C)** Transcranial direct current stimulation (tDCS) to PPC show no improvement of VWM in both the low and high capacity participants.

Participants viewed visual stimuli which consisted of 20 grayscale drawings of common objects (e.g., cat, fence; Rossion and Pourtois, 2004). In a 4 × 4 grid, five items were presented (500 ms), followed by a delay (750 ms), and the appearance of a single probe item. A total of 50 trials were presented. Participants made a new/old judgment, indicating whether the probe item was previously seen. Participant pressed the keys “o” or “n” to indicate whether the item or location was old or new (unspeeded), respectively; see Figure 4A.

### Transcranial Direct Current Stimulation

tDCS was applied to participants who were blinded to their tDCS condition. The anode was placed over right PPC (P4) and the cathode over the right contralateral cheek (CC). tDCS (1.5 mA, 10 min) was delivered through two 5 × 7 cm^2^ electrodes housed in saline-moistened sponges. The sham group had the electrodes placed over either the PFC or the PPC due to additional factors in the original experiment; see Figure 4B. Sham stimulation included 20 s of ramping up and down at the beginning and end of the stimulation time window.

### Analysis

The high and low VWM capacity groups were formed by median split based on OSpan scores: sham (high: 30.66, low: 11, *t*_(8)_ = −6.2, *p* < 0.00001), PPC (high: 30, low: 8.88, *t*_(8)_ = −6.2, *p* < 0.00001) the groups do not different in terms of age: high WMC (*t*_(8)_ = 0.05, *p* < 0.96), low WMC (*t*_(8)_ = 0, *p* < 1) or MMSE: high WMC (*t*_(8)_ = 0.6, *p* < 0.56), low WMC (*t*_(8)_ = 0.42, *p* < = 0.67). Groups included nine participants each.

## Results

A two-way ANOVA examined the effect of tDCS (Sham, PPC) and VWM capacity (low, high) on VWM accuracy; see Figure 4C. There was no main effect of tDCS (*F*_(1,32)_ = 0.56, *p* = 0.46, partial *η*^2^ = 0.01), or VWM capacity (*F*_(1,32)_ = 0.08, *p* = 0.78, partial *η*^2^ = 0.002) on VWM accuracy. No interactions approached significance (all *F*s < 1, *p*s > 0.46). The results of the ANOVA show that anodal tDCS to PPC did not alter VWM performance.

These data provide some insight as to whether the tDCS-linked VWM benefit observed in Experiment 1A could be entirely attributed to right PPC stimulation. Despite several paradigmatic differences between Experiments 1A and 1B, the data provide some confirmation that one session of right PPC stimulation is not sufficient to bolster VWM performance in these sorts of VWM tests probed by recognition. Important to note that behavioral performance did not reach ceiling (>90% accuracy) or floor (below chance) in this task as well; see Table 2.

**Table 2:** Accuracy (%) for each older adult group (low, high WM capacity) for the VWM task.

Measure	Group	Montage	Task Performance
%	Low	PPC-CC	73.3 (0.02)
		Sham	74.2 (0.03)
	High	PPC-CC	73.1 (0.02)
		Sham	70.8 (0.02)

*PPC-CC, posterior parietal cortex. Means (standard error of the mean)*.

## Experiment 2: Young Adults

Experiment 1A revealed a benefit of unilateral tDCS compared to bilateral frontal tDCS exclusively in the low VWM capacity group. Because younger adults show greater hemispheric lateralization than older adults, this raised the question of whether we might improve VWM performance in younger adults when applying a unilateral montage, or whether in this population heightening frontal activity via bilateral frontal tDCS. In essence, this was the inverse of our predictions for the healthy older adults. This experiment also extended our previous findings showing improved VWM in high VWM capacity participants after anodal or cathodal tDCS targeting the right posterior parietal lobe, whereas those with low VWM capacity showed impairment in both cases (Berryhill and Jones, 2012).

## Materials and Methods

A group of 36 self-reported right-handed younger adults (mean age: 21.22, SD: 2.38, 22 females) participated. The experimental procedures remained consistent with Experiment 1A except that the younger adult group did not perform the MOCA.

### Analysis

The high and low VWM capacity groups were formed by conducting a median split based on their Corsi Block Spatial Span scores (high: 12.22, low: 10, *t*_(35)_ = 6.4, *p* < 0.00001), the resulting groups were not different in terms of age (*p* = 0.68) there were a total of 18 participants in each group.

## Results

### LTM Task Results

**Figure 5:**
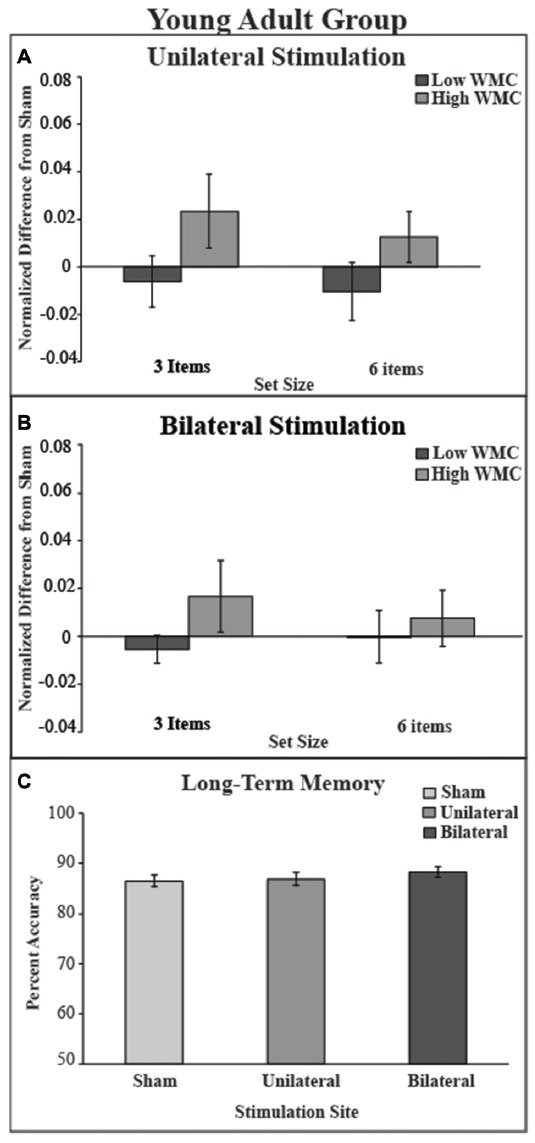
Behavioral outcome for younger adult group.** (A)** There was no benefit of stimulation in either low or high VWM capacity groups in the unilateral stimulation site. **(B)** There was no benefit of stimulation in the bilateral stimulation site. Values greater than zero reveal tDCS-related improvement in VWM performance. Error bars reflect the standard error. **(C)** In the LTM task there was no main effect of montage.

Difference indices were no different for either condition: unilateral (*M* = 0.01, SD = 0.02) and bilateral stimulation (*M* = 0.002, SD = 0.03; *t*_(35)_ = 1.6, *p* = 0.1). The tDCS montage did not significantly impact LTM task performance; see Figure 5C.

### VWM Task Results

The mixed model ANOVA included the factors of montage (unilateral, bilateral), VWM capacity (high, low), and set size (3, 6) revealed no significant main effects of stimulation (*F*_(1,34)_ = 0, *p* = 1, partial *η*^2^ = 0), set size (*F*_(1,34)_ = 0.30, *p* = 0.58, partial *η*^2^ = 0.01), or capacity: (*F*_(1,34)_ = 2.97, *p* = 0.09). No interactions were noted (all *p*s > 0.47). There was a non-significant numerical trend showing improved VWM performance in the high WM capacity group after *either* tDCS montage (see Table 3 and Figure 5).

**Table 3:** Difference indices (DI) and accuracy (%) for each younger adult group (low, high WM capacity) and set size (3, 6) in the VWM task.

Measure	Group	Montage	Set size 3	Set size 6	LTM
	Low	Uni	−0.006 (0.01)	−0.01 (0.01)	0.01 (0.01)
DI		Bi	−0.005 (0.005)	0.0 (0.01)	0.01 (0.01)
	High	Uni	0.023 (0.01)	0.007 (0.01)	−0.004 (0.01)
		Bi	0.016 (0.01)	0.01 (0.01)	0.01 (0.005)
	Low	Uni	84.8 (0.02)	67 (0.01)	86.7 (0.02)
%		Bi	85.3 (0.02)	68.9 (0.02)	87.1 (0.01)
		Sham	86.1 (0.03)	69 (0.02)	85.2 (0.02)
	High	Uni	87.9 (0.02)	72 (0.02)	87 (0.02)
		Bi	86.9 (0.03)	71.7 (0.03)	89.3 (0.01)
		Sham	84.9 (0.04)	70.6 (0.03)	87.7 (0.01)

*Uni, unilateral frontoparietal stimulation; Bi, bilateral frontal stimulation. Means (standard error of the mean)*.

#### Between Experiments Results

To permit comparison across the Experiments 1A–2, we combined the data and included the between-subjects factor of age (young, old).

### LTM Task

A mixed-effects measures ANOVA on retrieval accuracy with within-subject factors of montage (unilateral, bilateral, sham) and between-subject factors of age (young, old) confirmed superior performance in the young *adults* (*F*_(1,70)_ = 9.94, *p* = 0.002, partial *η*^2^ = 0.12). Neither the main effect of montage or the interaction approached significance (all *F*s < 1, *p*s > 18.

### VWM Task

A 2 (montage: unilateral, bilateral) × 2 (WM capacity: high, low) × 2 (set size: 3, 6) × 2 (age: young, old) mixed effects ANOVA using the DI data revealed a significant main effect of montage (*F*_(1,68)_ = 5.63, *p* = 0.02, partial *η*^2^ = 0.08) reflecting the VWM benefit of unilateral tDCS. No other main effects approached significance (Fs < 1, *p*s > 0.47). There was also an intriguing interaction of montage × age (*F*_(1,68)_ = 5.62, *p* = 0.02, partial *η*^2^ = 0.07) such that older adults garnered greater benefit from the unilateral montage compared to the younger adults; see Tables 1, 3. A significant two-way interaction of VWM capacity × age emerged (*F*_(1,68)_ = 6.56, *p* = 0.01, partial *η*^2^ = 0.09) revealing greater benefits in *high* VWM capacity young adults and *low* VWM capacity older adults. Of greatest interest was the significant three-way interaction of montage × VWM capacity × age (*F*_(1,68)_ = 9.12, *p* = 0.004, partial *η*^2^ = 0.12) again reflecting the greater benefit from the *unilateral* montage provided a VWM performance benefit to older adults with *low* VWM capacity (SS 3: 0.041 (SD: 0.073), SS 6: 0.043 (0.05)), and to younger adults with *high* VWM capacity (SS 3: 0.023 (SD: 0.06), SS 6: 0.01 (0.04)). No other interactions reached significance (montage × VWM capacity: *F*_(1,68)_ = 3.3, *p* = 0.07, montage × set size × age: *F*_(1,68)_ = 2.34, *p* = 0.14).

In summary, after collapsing across Experiments 1A and 2, the main effect of montage revealed a benefit of unilateral frontoparietal tDCS. The relationship is complex, as indicated by a significant three-way interaction of tDCS montage, VWM capacity, and age. Low WM capacity older adults and high WM capacity younger adults garnered WM benefit after unilateral frontoparietal tDCS.

## General Discussion

There is understandable interest in developing interventions that help to maintain cognitive performance that accompanies aging. Toward this end, tDCS and other neuromodulatory techniques show promise particularly when used in longitudinal paradigms. However, one challenge in developing tDCS for translational use involves the difficulty of optimizing a broad parameter space for each cognitive task without a complete understanding of the underlying mechanism(s). An additional, related challenge is that some individual differences predict an individual’s behavioral response to tDCS, but which ones are consequential and why remains unclear. Finally, a challenge for tDCS application has been the enormous parameter space and little clear optimization, and few experimental approaches relying on theoretically grounded alternative hypotheses.

Given these unknowns, we initiated the current experiments to test whether healthy older adults would receive greater VWM benefit from a frontoparietal tDCS montage that was hypothesized to restore a more lateralized pattern of VWM-related brain activity, or from a bifrontal montage hypothesized to support compensatory bilateral frontal activity. The data showed that the healthy older adults’ performance on the VWM change detection task improved when the unilateral tDCS montage was applied to restore a more youthful pattern of brain activations. However, this VWM performance benefit was limited to the older adults with lower WM capacity. The high WM capacity group of older adults showed no notable change in VWM or LTM performance for either tDCS montage. Next, we reanalyzed existing data to ensure that it was right unilateral stimulation that was driving our significant main-effect of stimulation. The data showed that stimulation to PPC in older adults showed no benefit of stimulation following a single session of tDCS when compared to a control group. This supports our findings that restoration of a more youthful pattern of brain activity is beneficial to older adults with WM capacity. We also tested high and low WM capacity younger adults to see if there were differential benefits associated with either tDCS montage. Here, the logic was that reinforcing their more lateralized pattern of activity might support VWM performance, or alternatively, by supporting greater bilateral frontal activity VWM performance might improve. The data showed that there was a VWM benefit associated with the unilateral montage but in the high WM capacity group. Instead of clearly supporting a uniform tDCS protocol to enhance WM across participants, or across participants with a particular WM capacity, these data highlight the difficulty of anticipating how individual differences affect responsiveness to tDCS. The data confirm that age and WM capacity are important factors that should be considered when designing tDCS-linked experiments or therapeutic interventions.

A serious challenge in interpreting several critical meta-analyses of tDCS-linked effects on WM (Horvath et al., 2015a,b; Mancuso et al., 2016) is that few studies include VWM capacity in analyses. We reliably and regularly observe nearly equal and opposite responses to a given tDCS protocol when we take into consideration a single difference: WM capacity. There is a growing literature indicating that what participants bring to the table cognitively and morphologically contributes to the predicted outcomes (Kim et al., 2014; Krause and Cohen Kadosh, 2014; Russell et al., 2014; Li et al., 2015; London and Slagter, 2015). What is not clear is why a factor such as working WM has this predictive power. The importance of individual differences should be included among the key variables of interest in neuromodulatory research (reviewed in Dedoncker et al., 2016).

Several years ago, we were surprised to find that *either* anodal or cathodal tDCS targeting the posterior parietal improved VWM performance in older adults with more education (Berryhill and Jones, 2012). Those with less education showed nearly equal and opposite effects, such that their VWM performance was worse after tDCS. Here, we find that WM capacity and age interact, as well. Although these measures of group differences are different, we think they are related. Putting these observations together suggests that the way low and high WM capacity older adults strategically approach WM tasks involves different patterns of neural activity (Gray et al., 2003; Osaka et al., 2004; Lee et al., 2006). We have some data supporting this speculation regarding strategy. Previously, we showed that high capacity VWM participants could benefit from either anodal or cathodal tDCS targeting the posterior parietal lobe, but only when the task is difficult (Jones and Berryhill, 2012). Adding financial reward and feedback to a verbal WM task and applying anodal tDCS over the left PFC helped the low and high capacity young adults alike (Jones et al., 2015a). These data again point toward a differential usage of frontoparietal networks during WM tasks.

Finally, what is striking about these significant individual differences in single-session per condition designs is that all older adults in a longitudinal design targeting either the PFC, the PPC, or alternating between both sites, showed WM benefits (Jones et al., 2015b). Apart from the importance of individual differences in WM capacity, and age, the number of sessions in the protocol matter. For researchers interested in using tDCS to study structure-function relationships this may be a more serious concern than for researchers interested in translational applications because at least the longitudinal designs are consistently beneficial.

## Limitations

We acknowledge that to be more generalizable we would want to include a wider variety of WM tasks. A limitation of this work is that the present study involved testing only a single VWM task. We also tested VWM alone and implemented a right-lateralized unilateral tDCS protocol. To understand whether verbal WM performance responds similarly a left-lateralized tDCS experiment is needed. Despite these limitations, our observation that the behavioral consequences of tDCS depend on who is doing the participating and where the stimulation is targeting. These data do suggest that the strategy involved in conducting the task will be important to specify in going forward. We also acknowledge that our hypotheses about effects are derived from advances in current modeling which has validated success in predicting the regions affected by tDCS (Bikson et al., 2009; for a recent review see Reinhart et al., 2017) rather than from direct measurements of tDCS using neuroimaging in our own participants. A few studies in which online tDCS effects were measured using fMRI found current modeling to be consistent with contemporaneous measures of brain activation (Zheng et al., 2011; Meinzer et al., 2012, 2013; Alekseichuk et al., 2016). Although it would be superior to pair tDCS and fMRI to validate the pattern of altered activity in each individual and each session, this would impose technical and financial challenges. Finally, Experiment 1B suffers from low power and future work will be needed to fully characterize interactions between various frontoparietal regions, and tDCS as measured by behavior. These gaps are important to note as we continue to progress in developing interventions and protocols including tDCS that benefit as many people as possible.

## Author Contributions

HA, FG and MEB conceived and designed the experiment. HA and FG collected behavioral-tDCS data and analyzed behavioral data. HA and MEB contributed to writing and revising the manuscript. KTJ and JAS conceived, designed, collected behavioral-tDCS data for Experiment 1B.

## Conflict of Interest Statement

The authors declare that the research was conducted in the absence of any commercial or financial relationships that could be construed as a potential conflict of interest.

## References

[B1] Alekseichuk I. Diers K. Paulus W. Antal A. (2016). Transcranial electrical stimulation of the occipital cortex during visual perception modifies the magnitude of BOLD activity: a combined tES-fMRI approach. Neuroimage 140, 110–117. 10.1016/j.neuroimage.2015.11.034 26608246

[B2] Anderson N. D. Iidaka T. Cabeza R. Kapur S. McIntosh A. R. Craik F. I. M. (2000). The effects of divided attention on encoding- and retrieval-related brain activity: a PET study of younger and older adults. J. Cogn. Neurosci. 12, 775–792. 10.1162/089892900562598 11054920

[B3] Ansado J. Monchi O. Ennabil N. Faure S. Joanette Y. (2012). Load-dependent posterior-anterior shift in aging in complex visual selective attention situations. Brain Res. 1454, 14–22. 10.1016/j.brainres.2012.02.061 22483790

[B4] Ansaldo A. I. Ghazi-Saidi L. Adrover-Roig D. (2015). Interference control in elderly bilinguals: appearances can be misleading. J. Clin. Exp. Neuropsychol. 37, 455–470. 10.1080/13803395.2014.990359 25641572

[B5] Au J. Buschkuehl M. Duncan G. J. Jaeggi S. M. (2016). There is no convincing evidence that working memory training is NOT effective: a reply to Melby-Lervåg and Hulme (2015). Psychon. Bull. Rev. 23, 331–337. 10.3758/s13423-015-0967-4 26518308

[B7] Berryhill M. E. (2012). Insights from neuropsychology: pinpointing the role of the posterior parietal cortex in episodic and working memory. Front. Integr. Neurosci. 6:31. 10.3389/fnint.2012.00031 22701406 PMC3371666

[B10] Berryhill M. E. (2017). Longitudinal tDCS: consistency across working memory training studies. AIMS Neurosci. 4, 71–86. 10.3934/neuroscience.2017.2.71

[B6] Berryhill M. E. Jones K. T. (2012). tDCS selectively improves working memory in older adults with more education. Neurosci. Lett. 521, 148–151. 10.1016/j.neulet.2012.05.074 22684095

[B8] Berryhill M. E. Olson I. R. (2008a). Is the posterior parietal lobe involved in working memory retrieval? Evidence from patients with bilateral parietal lobe damage. Neuropsychologia 46, 1775–1786. 10.1016/j.neuropsychologia.2008.03.005 18439630 PMC2494709

[B11] Berryhill M. E. Olson I. R. (2008b). The right parietal lobe is critical for visual working memory. Neuropsychologia 46, 1767–1774. 10.1016/j.neuropsychologia.2008.01.009 18308348 PMC2441642

[B12] Berryhill M. E. Peterson D. J. Jones K. T. Stephens J. A. (2014). Hits and misses: leveraging tDCS to advance cognitive research. Front. Psychol. 5:800. 10.3389/fpsyg.2014.00800 25120513 PMC4111100

[B9] Berryhill M. E. Wencil E. B. Branch Coslett B. Olson I. R. (2010). A selective working memory impairment after transcranial direct current stimulation to the right parietal lobe. Neurosci. Lett. 479, 312–316. 10.1016/j.neulet.2010.05.087 20570713 PMC2902585

[B13] Bikson M. Datta A. Elwassif M. (2009). Establishing safety limits for transcranial direct current stimulation. Clin. Neurophysiol. 120, 1033–1034. 10.1016/j.clinph.2009.03.018 19394269 PMC2754807

[B15] Cabeza R. (2002). Hemispheric asymmetry reduction in older adults: the HAROLD model. Psychol. Aging 17, 85–100. 10.1037//0882-7974.17.1.85 11931290

[B17] Cabeza R. Daselaar S. M. Dolcos F. Prince S. E. Budde M. Nyberg L. (2004). Task-independent and task-specific age effects on brain activity during working memory, visual attention and episodic retrieval. Cereb. Cortex 14, 364–375. 10.1093/cercor/bhg133 15028641

[B16] Cabeza R. Nyberg L. (2000). Imaging cognition II: an empirical review of 275 PET and fMRI studies. J. Cogn. Neurosci. 12, 1–47. 10.1162/08989290051137585 10769304

[B18] Carp J. Gmeindl L. Reuter-Lorenz P. A. (2010). Age differences in the neural representation of working memory revealed by multi-voxel pattern analysis. Front. Hum. Neurosci. 4:217. 10.3389/fnhum.2010.00217 21151373 PMC2996172

[B19] Chan M. Y. Park D. C. Savalia N. K. Petersen S. E. Wig G. S. (2014). Decreased segregation of brain systems across the healthy adult lifespan. Proc. Natl. Acad. Sci. U S A 111, E4997–E5006. 10.1073/pnas.1415122111 25368199 PMC4246293

[B20] Cotelli M. Manenti R. Petesi M. Brambilla M. Cosseddu M. Zanetti O.. (2014). Treatment of primary progressive aphasias by transcranial direct current stimulation combined with language training. J. Alzheimers Dis. 39, 799–808. 10.3233/JAD-131427 24296814

[B21] Craik F. I. M. Luo L. Sakuta Y. (2010). Effects of aging and divided attention on memory for items and their contexts. Psychol. Aging 25, 968–979. 10.1037/a0020276 20973605

[B22] Davis S. W. Dennis N. A. Daselaar S. M. Fleck M. S. Cabeza R. (2008). Que PASA? The posterior-anterior shift in aging. Cereb. Cortex 18, 1201–1209. 10.1093/cercor/bhm155 17925295 PMC2760260

[B23] Dedoncker J. Brunoni A. R. Baeken C. Vanderhasselt M. (2016). A systematic review and meta-analysis of the effects of transcranial direct current stimulation (tDCS) over the dorsolateral prefrontal cortex in healthy and neuropsychiatric samples: influence of stimulation parameters. Brain Stimul. 9, 501–517. 10.1016/j.brs.2016.04.006 27160468

[B25] Dennis N. A. Hayes S. M. Prince S. E. Madden D. J. Huettel S. A. Cabeza R. (2008). Effects of aging on the neural correlates of successful item and source memory encoding. J. Exp. Psychol. Learn. Mem. Cogn. 34, 791–808. 10.1037/0278-7393.34.4.791 18605869 PMC2752883

[B24] Dennis N. A. Peterson K. M. (2012). Neural correlates mediating age differences in episodic memories: evidence from bold contrasts and connectivity analyses. Psychologia 55, 112–130. 10.2117/psysoc.2012.112

[B26] D’Esposito M. Postle B. R. Rypma B. (2000). Prefrontal cortical contributions to working memory: evidence from event-related fMRI studies. Exp. Brain Res. 133, 3–11. 10.1007/s002210000395 10933205

[B27] Gandiga P. C. Hummel F. C. Cohen L. G. (2006). Transcranial DC stimulation (tDCS): a tool for double-blind sham-controlled clinical studies in brain stimulation. Clin. Neurophysiol. 117, 845–850. 10.1016/j.clinph.2005.12.003 16427357

[B28] Gill J. Shah-Basak P. P. Hamilton R. (2015). It’s the thought that counts: examining the task-dependent effects of transcranial direct current stimulation on executive function. Brain Stimul. 8, 253–259. 10.1016/j.brs.2014.10.018 25465291

[B30] Grady C. L. (1996). Age-related changes in cortical blood flow activation during perception and memory. Anna. N Y Acad. Sci. 777, 14–21. 10.1111/j.1749-6632.1996.tb34396.x 8624077

[B29] Grady J. R. (2000). Functional brain imaging and age-related changes in cognition. Biol. Psychol. 54, 259–281. 10.1016/s0301-0511(00)00059-4 11035226

[B31] Gray J. R. Chabris C. F. Braver T. S. (2003). Neural mechanisms of general fluid intelligence. Nat. Neurosci. 6, 316–322. 10.1038/nn1014 12592404

[B32] Grossman M. Cooke A. DeVita C. Alsop D. Detre J. Chen W.. (2002). Age-related changes in working memory during sentence comprehension: an fMRI study. Neuroimage 15, 302–317. 10.1006/nimg.2001.0971 11798267

[B33] Gutchess A. H. Welsh R. C. Hedden T. Bangert A. Minear M. Liu L. L.. (2005). Aging and the neural correlates of successful picture encoding: frontal activations compensate for decreased medial-temporal activity. J. Cogn. Neurosci. 17, 84–96. 10.1162/0898929052880048 15701241

[B35] Horvath J. C. Forte J. D. Carter O. (2015a). Evidence that transcranial direct current stimulation (tDCS) generates little-to-no reliable neurophysiologic effect beyond MEP amplitude modulation in healthy human subjects: a systematic review. Neuropsychologia 66, 213–236. 10.1016/j.neuropsychologia.2014.11.021 25448853

[B36] Horvath J. C. Forte J. D. Carter O. (2015b). Quantitative review finds no evidence of cognitive effects in healthy populations from single-session transcranial direct current stimulation (tDCS). Brain Stimul. 8, 535–550. 10.1016/j.brs.2015.01.400 25701175

[B37] Hsu T. Y. Juan C. H. Tseng P. (2016). Individual differences and state-dependent responses in transcranial direct current stimulation. Front. Hum. Neurosci. 10:643. 10.3389/fnhum.2016.00643 28066214 PMC5174116

[B80] Iachini T. Iavarone A. Senese V. P. Ruotolo F. Ruggiero G. (2009). Visuospatial memory in healthy elderly, AD and MCI: a review. Curr. Aging Sci. 2, 43–59. 10.2174/1874609810902010043 20021398

[B38] Iidaka T. Okada T. Murata T. Omori M. Kosaka H. Sadato N.. (2002). Age-related differences in the medial temporal lobe responses to emotional faces as revealed by fMRI. Hippocampus 12, 352–362. 10.1002/hipo.1113 12099486

[B39] Jo J. M. Kim Y. H. Ko M. H. Ohn S. H. Joen B. Lee K. H. (2009). Enhancing the working memory of stroke patients using tDCS. Am. J. Phys. Med. Rehabil. 88, 404–409. 10.1097/phm.0b013e3181a0e4cb 19620953

[B40] Jockwitz C. Caspers S. Lux S. Jütten K. Schleicher A. Eickhoff S. B.. (2017). Age- and function-related regional changes in cortical folding of the default mode network in older adults. Brain Struct. Funct. 222, 83–99. 10.1007/s00429-016-1202-4 26943919

[B41] Jones K. T. Berryhill M. E. (2012). Parietal contributions to visual working memory depend on task difficulty. Front. Psychiatry 3:81. 10.3389/fpsyt.2012.00081 22973241 PMC3437464

[B42] Jones K. T. Gözenman F. Berryhill M. E. (2015a). The strategy and motivational influences on the beneficial effect of neurostimulation: a tDCS and fNIRS study. Neuroimage 105, 238–247. 10.1016/j.neuroimage.2014.11.012 25462798 PMC4262534

[B43] Jones K. T. Stephens J. A. Alam M. Bikson M. Berryhill M. E. (2015b). Correction: longitudinal neurostimulation in older adults improves working memory. PLoS One 10:e0129751. 10.1371/journal.pone.0129751 25849358 PMC4388845

[B45] Juan C.-H. Tseng P. Hsu T.-Y. (2017). Elucidating and modulating the neural correlates of visuospatial working memory via noninvasive brain stimulation. Curr. Dir. Psychol. Sci. 26, 165–173. 10.1177/0963721416677095

[B46] Katz B. Au J. Buschkuehl M. Abagis T. Zabel C. Jaeggi S. M.. (2017). Individual differences and long-term consequences of tDCS-augmented cognitive training. J. Cogn. Neurosci. 29, 1498–1508. 10.1162/jocn_a_01115 28253083

[B47] Kim J. H. Kim D. W. Chang W. H. Kim Y. H. Kim K. Im C. H. (2014). Inconsistent outcomes of transcranial direct current stimulation may originate from anatomical differences among individuals: electric field simulation using individual MRI data. Neurosci. Lett. 564, 6–10. 10.1016/j.neulet.2014.01.054 24508704

[B48] Krause B. Cohen Kadosh R. (2014). Not all brains are created equal: the relevance of individual differences in responsiveness to transcranial electrical stimulation. Front. Syst. Neurosci. 8:25. 10.3389/fnsys.2014.00025 24605090 PMC3932631

[B49] Lee K. H. Choi Y. Y. Gray J. R. Cho S. H. Chae J. H. Lee S.. (2006). Neural correlates of superior intelligence: stronger recruitment of posterior parietal cortex. Neuroimage 29, 578–586. 10.1016/j.neuroimage.2005.07.036 16122946

[B50] Li L. M. Uehara K. Hanakawa T. (2015). The contribution of interindividual factors to variability of response in transcranial direct current stimulation studies. Front. Cell. Neurosci. 9:181. 10.3389/fncel.2015.00181 26029052 PMC4428123

[B52] London R. E. Slagter H. A. (2015). Effects of transcranial direct current stimulation over left dorsolateral pFC on the attentional blink depend on individual baseline performance. J. Cogn. Neurosci. 27, 2382–2393. 10.1162/jocn_a_00867 26284996

[B53] Madden D. J. Turkington T. G. Provenzale J. M. Denny L. L. Langley L. K. Hawk T. C.. (2002). Aging and attentional guidance during visual search: functional neuroanatomy by positron emission tomography. Psychol. Aging 17, 24–43. 10.1037/0882-7974.17.1.24 11931285 PMC1831840

[B54] Mancuso L. E. Ilieva I. P. Hamilton R. H. Farah M. J. (2016). Does transcranial direct current stimulation improve healthy working memory? a meta-analytic review. J. Cogn. Neurosci. 28, 1063–1089. 10.1162/jocn_a_00956 27054400

[B55] Meinzer M. Antonenko D. Lindenberg R. Hetzer S. Avirame K. Flaisch T.. (2012). Electrical brain stimulation improves cognitive performance by modulating functional connectivity and task-specific activation. J. Neurosci. 32, 1859–1866. 10.1523/JNEUROSCI.4812-11.2012 22302824 PMC6703352

[B56] Meinzer M. Lindenberg R. Antonenko D. Flaisch T. Flöel A. (2013). Anodal transcranial direct current stimulation temporarily reverses age-associated cognitive decline and functional brain activity changes. J. Neurosci. 33, 12470–12478. 10.1523/JNEUROSCI.5743-12.2013 23884951 PMC6618670

[B57] Meulenbroek O. Petersson K. M. Voermans N. Weber B. Fernández G. (2004). Age differences in neural correlates of route encoding and route recognition. Neuroimage 22, 1503–1514. 10.1016/j.neuroimage.2004.04.007 15275907

[B59] Nasseri P. Nitsche M. A. Ekhtiari H. (2015). A framework for categorizing electrode montages in transcranial direct current stimulation. Front. Hum. Neurosci. 9:54. 10.3389/fnhum.2015.00054 25705188 PMC4319395

[B60] Nuechterlein K. H. Green M. F. Kern R. S. Baade L. E. Barch D. M. Cohen J. D.. (2008). The MATRICS consensus cognitive battery, part 1: test selection, reliability, and validity. Am. J. Psychiatry 165, 203–213. 10.1176/appi.ajp.2007.07010042 18172019

[B61] Nyberg L. Sandblom J. Jones S. Neely A. S. Petersson K. M. Ingvar M.. (2003). Neural correlates of training-related memory improvement in adulthood and aging. Proc. Natl. Acad. Sci. U S A 100, 13728–13733. 10.1073/pnas.1735487100 14597711 PMC263881

[B62] Olson I. R. Berryhill M. (2009). Some surprising findings on the involvement of the parietal lobe in human memory. Neurobiol. Learn. Mem. 91, 155–165. 10.1016/j.nlm.2008.09.006 18848635 PMC2898273

[B63] Osaka N. Osaka M. Kondo H. Morishita M. Fukuyama H. Shibasaki H. (2004). The neural basis of executive function in working memory: an fMRI study based on individual differences. Neuroimage 21, 623–631. 10.1016/j.neuroimage.2003.09.069 14980565

[B65] Park S. Gooding D. C. (2014). Working memory impairement as an endophenotypic marker of a schizophrenia diathesis. Schizophr. Res. Cogn. 1, 127–136. 10.1016/j.scog.2014.09.005 25414816 PMC4234058

[B66] Park S. Püschel J. Sauter B. H. Rentsch M. Hell D. (2003). Visual object working memory function and clinical symptoms in schizophrenia. Schizophr. Res. 59, 261–268. 10.1016/s0920-9964(02)00209-8 12414083

[B64] Park D. C. Reuter-Lorenz P. (2009). The adaptive brain: aging and neurocognitive scaffolding. Annu. Rev. Psychol. 60, 173–196. 10.1146/annurev.psych.59.103006.093656 19035823 PMC3359129

[B67] Pendlebury S. T. Klaus S. P. Mather M. de Brito M. Wharton R. M. (2015). Routine cognitive screening in older patients admitted to acute medicine: abbreviated mental test score (AMTS) and subjective memory complaint versus Montreal Cognitive Assessment and IQCODE. Age Ageing 44, 1000–1005. 10.1093/ageing/afv134 26464420 PMC4621235

[B68] Puri R. Hinder M. R. Canty A. J. Summers J. J. (2016). Facilitatory non-invasive brain stimulation in older adults: the effect of stimulation type and duration on the induction of motor cortex plasticity. Exp. Brain Res. 234, 3411–3423. 10.1007/s00221-016-4740-3 27450080

[B69] Reinhart R. M. G. Cosman J. D. Fukuda K. Woodman G. F. (2017). Using transcranial direct-current stimulation (tDCS) to understand cognitive processing. Atten. Percept. Psychophys. 79, 3–23. 10.3758/s13414-016-1224-2 27804033 PMC5539401

[B70] Reuter-Lorenz P. A. Cappell K. A. (2008). Neurocognitive aging and the compensation hypothesis. Curr. Dir. Psychol. Sci. 17, 177–182. 10.1111/j.1467-8721.2008.00570.x

[B71] Reuter-Lorenz P. A. Park D. C. (2014). How does it STAC up? Revisiting the scaffolding theory of aging and cognition. Neuropsychol. Rev. 24, 355–370. 10.1007/s11065-014-9270-9 25143069 PMC4150993

[B72] Reuter-Lorenz P. A. Sylvester C.-Y. C. (2005). “The cognitive neuroscience of working memory and aging,” in Cognitive Neuroscience of Aging: Linking Cognitive and Cerebral Aging, eds Cabeza R. Nyberg L. Park D. (New York, NY: Oxford University Press), 186–217.

[B73] Richmond L. L. Wolk D. Chein J. Olson I. R. (2014). Transcranial direct current stimulation enhances verbal working memory training performance over time and near transfer outcomes. J. Cogn. Neurosci. 26, 2443–2454. 10.1162/jocn_a_00657 24742190

[B74] Rossion B. Pourtois G. (2004). Revisiting snodgrass and vanderwart’s object pictorial set: the role of surface detail in basic-level object recognition. Perception 33, 217–236. 10.1068/p5117 15109163

[B75] Russell M. Goodman T. Wang Q. Groshong B. Lyeth B. G. (2014). Gender differences in current received during transcranial electrical stimulation. Front. Psychiatry 5:104. 10.3389/fpsyt.2014.00104 25177301 PMC4133690

[B76] Rypma B. D’Esposito M. (2000). Isolating the neural mechanisms of age-related changes in human working memory. Nat. Neurosci. 3, 509–515. 10.1038/74889 10769393

[B77] Sandrini M. Umiltà C. Rusconi E. (2011). The use of transcranial magnetic stimulation in cognitive neuroscience: a new synthesis of methodological issues. Neurosci. Biobehav. Rev. 35, 516–536. 10.1016/j.neubiorev.2010.06.005 20599555

[B78] Schneider-Garces N. J. Gordon B. A. Brumback-Peltz C. R. Shin E. Lee Y. Sutton B. P.. (2010). Span, CRUNCH, and beyond: working memory capacity and the aging brain. J. Cogn. Neurosci. 22, 655–669. 10.1162/jocn.2009.21230 19320550 PMC3666347

[B79] Stephens J. A. Berryhill M. E. (2016). Older adults improve on everyday tasks after working memory training and neurostimulation. Brain Stimul. 9, 553–559. 10.1016/j.brs.2016.04.001 27178247 PMC4957521

[B81] Todd J. J. Marois R. (2004). Capacity limit of visual short-term memory in human posterior parietal cortex. Nature 428, 751–754. 10.1038/nature02466 15085133

[B82] Todd J. J. Marois R. (2005). Posterior parietal cortex activity predicts individual differences in visual short-term memory capacity. Cogn. Affect. Behav. Neurosci. 5, 144–155. 10.3758/cabn.5.2.144 16180621

[B84] Tseng P. Chang C. Chiau H. Liang W. K. Liu C. L. Hsu T. Y.. (2013). The dorsal attentional system in oculomotor learning of predictive information. Front. Hum. Neurosci. 7:404. 10.3389/fnhum.2013.00404 23935573 PMC3731626

[B85] Unsworth N. Heitz R. P. Schrock J. C. Engle R. W. (2005). An automated version of the operation span task. Behav. Res. Methods 37, 498–505. 10.3758/bf03192720 16405146

[B86] Vogel E. K. Machizawa M. G. (2004). Neural activity predicts individual differences in visual working memory capacity. Nature 428, 748–751. 10.1038/nature02447 15085132

[B87] Vogel E. K. McCullough A. W. Machizawa M. G. (2005). Neural measures reveal individual differences in controlling access to working memory. Nature 438, 500–503. 10.1038/nature04171 16306992

[B101] Wu Y. J. Tseng P. Chang C. F. Pai M. C. Hsu K. S. Lin C. C.. (2014). Modulating the interference effect on spatial working memory by applying transcranial direct current stimulation over the right dorsolateral prefrontal cortex. Brain Cogn. 91, 87–94. 10.1016/j.bandc.2014.09.002 25265321

[B88] Wu Y. J. Tseng P. Huang H. W. Hu J. F. Juan C. H. Hsu K. S.. (2016). The facilitative effect of transcranial direct current stimulation on visuospatial working memory in patients with diabetic polyneuropathy: a pre-post sham-controlled study. Front. Hum. Neurosci. 10:479. 10.3389/fnhum.2016.00479 27733822 PMC5039168

[B89] Xu Y. Chun M. M. (2006). Dissociable neural mechanisms supporting visual short-term memory for objects. Nature 440, 91–95. 10.1038/nature04262 16382240

[B90] Zheng X. Alsop D. C. Schlaug G. (2011). Effects of transcranial direct current stimulation (tDCS) on human regional cerebral blood flow. Neuroimage 58, 26–33. 10.1016/j.neuroimage.2011.06.018 21703350 PMC3155947

